# Sleep disturbances as early indicators of incident psychiatric disease

**DOI:** 10.1007/s00406-026-02332-9

**Published:** 2026-08-11

**Authors:** Jie Guo, Linda L. Magnusson Hanson, Torbjörn Åkerstedt, Anna Karin Hedström

**Affiliations:** 1 Department of Nutrition and Health, China Agricultural University, Beijing, China; 2 Department of Psychology, Stockholm University, Stockholm, Sweden; 3 Department of Psychology, Stockholm University, Stockholm, Sweden; 4 Department of Clinical Neuroscience, Karolinska Institutet, 171 76 Stockholm, Sweden

**Keywords:** Sleep disturbances, Insomnia, Fatigue, Incident psychiatric disease, Longitudinal

## Abstract

**Purpose:**

Sleep disturbances are common in individuals with psychiatric diseases and may emerge years before diagnosis. It remains unclear whether they reflect modifiable risk factors or early manifestations of psychiatric illness. We aimed to investigate how different sleep dimensions relate to the risk of subsequent psychiatric illness, and how sleep symptoms evolve before and after diagnosis.

**Methods:**

We analyzed data from 24,038 participants in the Swedish Longitudinal Occupational Survey of Health (2010–2018), linked to national health registers. Associations between baseline sleep characteristics and incident psychiatric diagnoses were estimated using Cox proportional hazards models. Symptom trajectories were modelled using non-linear mixed-effects models on a backward timescale centered at diagnosis.

**Results:**

A total of 716 participants (2.9%) were diagnosed with a psychiatric disorder during follow-up. There was a U-shaped association between sleep duration and incident psychiatric illness. All sleep-related symptoms were associated with increased risk, with the strongest association observed for mental fatigue (HR 2.09, 95% CI 1.77–2.48). Insufficient sleep and sleep-disordered breathing were more common among those who later developed psychiatric illness, with differences remaining relatively stable over time. In contrast, other sleep complaints, such as insomnia symptoms and fatigue, progressively worsened prior to diagnosis and improved thereafter, although levels remained elevated compared to unaffected individuals. Long sleep duration and delayed wake-up times were exceptions, showing continued worsening post-diagnosis.

**Conclusion:**

Gradually intensifying sleep disturbances may reflect emerging psychiatric vulnerability consistent with a prodromal phase. Where repeated monitoring is feasible, these symptoms could serve as early warning signs to guide preventive efforts.

**Supplementary Information:**

The online version contains supplementary material available at https://doi.org/10.1007/s00406-026-02332-9.

## Background

Sleep disturbances are common in individuals with psychiatric disorders and often precede clinical diagnosis by months or even years [1–4]. Epidemiological studies have linked sleep problems, including insomnia, fatigue, circadian disruption, and sleep-disordered breathing, to a broad range of psychiatric outcomes, such as depression, anxiety, and psychotic disorders [1–4]. However, it remains unclear whether these disturbances serve as modifiable risk factors or represent early symptoms of an evolving psychiatric condition [5–7].

While much of the existing literature has focused on isolated sleep measures or specific diagnostic categories, psychiatric disorders frequently share overlapping features such as mood instability, altered arousal regulation, and impaired stress responses, all of which can influence or be influenced by sleep [8–10]. Furthermore, sleep–wake dysregulation and impaired daytime functioning, manifested as persistent fatigue or mental fatigue, may reflect early neurobiological alterations that precede overt psychiatric symptoms [11, 12].

Prospective data on how multiple dimensions of sleep evolve in the lead-up to psychiatric illness remain scarce. In particular, little is known about whether long-term patterns of sleep disturbance, changes in sleep timing, or daytime symptoms differ in individuals who later develop psychiatric disorders compared to those who remain unaffected. Understanding these trajectories could help clarify the temporal dynamics of sleep–psychiatric relationships and improve early detection strategies.

In this study, we used repeated measurements from a large Swedish cohort to investigate if long-term patterns of sleep characteristics differ in individuals who develop psychiatric illness compared to those who remain unaffected.

## Methods

This study utilized data from the Swedish Longitudinal Occupational Survey of Health (SLOSH) [13], an ongoing cohort study initiated in 2006. The cohort comprises individuals sampled through the Swedish Work Environment Surveys (SWES), which target employed persons aged 16–64 years. Follow-up data have been collected biennially using postal questionnaires up to and including 2020. Participants received one of two questionnaire formats depending on employment status, either for those currently employed or for individuals working part-time or not at all. Both forms included consistent core items covering sleep habits, health indicators, and sociodemographic characteristics.

For this study, we focused on data from waves 3 through 7 (2010–2018), as these waves provided detailed and consistent assessments of sleep-related variables. At each wave, participants self-reported on a range of health, lifestyle, and sleep behaviors. Participation in the study was considered to reflect informed consent, as per Swedish ethical standards, through the return of completed questionnaires. The response rate decreased slightly over time, from 57% in 2010 to 48% in 2018. Compared with non-respondents, participants were more often women, born in Sweden, older, and more likely to hold a university degree.

Through linkage with Swedish national health registers using unique personal identity numbers, we obtained diagnostic data for psychiatric disorders (ICD codes F00-F99) from the National Patient Register and the Cause of Death Register. Among the 29,651 respondents who participated in any of the waves 3–7, we excluded individuals with an existing psychiatric diagnosis prior to baseline (*n* = 2781) and those lacking responses to all sleep-related items (*n* = 1832). The resulting sample comprised 24,038 participants (eFigure S1).

To examine longitudinal changes in sleep variables, we restricted trajectory-based analyses to individuals who had provided data at three or more waves between 2010 and 2018. A total of 16,845 participants met this inclusion criterion. Due to staggered introduction of certain sleep items and variable response rates across waves, the number of participants available for specific longitudinal models ranged from 15,035 (physical exhaustion) to 16,748 (sleep sufficiency). The study was approved by the Regional Ethics Review Board in Stockholm.

### Definition of sleep related items

Participants reported their typical bedtimes and wake-up times separately for workdays and days off. These data were used to estimate average weekly sleep duration, which was then analyzed as a continuous variable as well as categorized as short (< 7 h), normal (7–8 h), or long sleep (> 8 h). Sleep timing patterns were analyzed as continuous variables. To assess intra-week variability, we calculated social jetlag as the difference in mid-time sleep between workdays and non-workdays, an indicator of circadian misalignment.

Sleep-related symptoms were measured using items from the Karolinska Sleep Questionnaire [14], which assess both nighttime disturbances and daytime impairments. Participants rated the frequency of symptoms including difficulties falling asleep, repeated awakenings, premature awakenings, difficulty waking up, non-restorative sleep, snoring, sleep apnea, as well as persistent fatigue, physical exhaustion, and mental fatigue. Each symptom was scored on a six-point ordinal scale: (1) never, (2) rarely/single occasion, (3) sometimes/a few times a month, (4) frequently/1–2 times/week, (5) mostly/3–4 times/week, and (6) always/5 times or more per week. Nighttime symptoms were considered present if reported at least three times per week (responses 5–6), and daytime symptoms were coded as present if they occurred twice a week or more. Nighttime insomnia was defined as the presence of at least one of the following occurring three or more times per week: difficulties falling asleep, repeated awakenings, or premature awakenings. In addition, participants rated their subjective sleep sufficiency on a five-point scale, which was dichotomized into sufficient (scores 1–3) or insufficient (scores 4–5) sleep.

### Statistical analysis

Descriptive statistics were used to characterize the study population. Categorical variables were described with counts and proportions, while continuous variables were summarized using means and standard deviations (SD).

To investigate associations between sleep characteristics and the development of incident psychiatric disorder, we employed Cox proportional hazards regression models. Participants were followed from the date of their baseline questionnaire until the earliest occurrence of psychiatric diagnosis, death, loss to follow-up, or end of follow-up in 2018. The assumption of proportional hazard was tested using Schoenfeld residuals and found to be satisfied in all models. All Cox models were adjusted for a range of baseline covariates, selected based on prior research and clinical relevance. These included age, sex, educational attainment (university vs. no university), smoking status (current vs. non-smoker), physical activity level (low vs. high), and body mass index categorized as underweight, normal weight, overweight, or obese according to WHO standards. Additional adjustments were made for regular use of sleep medication (yes/no), habitual sleep duration (< 7, 7–8, > 8 h) and co-morbidities, including (cardiovascular disease ICD I00-I99, cancer ICD C00-C97, and neurological conditions ICD G00-G99).

To examine longitudinal patterns in sleep symptoms before and after psychiatric diagnosis, we applied linear mixed-effects models among participants with at least three repeated sleep assessments. Time was modeled on a backward scale, where 0 represented the year of diagnosis for those with psychiatric disease, or the end of follow-up for those without. The models included fixed effects for psychiatric disease status, time, and their interaction, enabling us to estimate diverging sleep trajectories and symptom evolution relative to diagnosis. Random effects included a random intercept and slope for time at the individual level. We compared rates of change in sleep variables between participants who developed a psychiatric disease and those who did not and further examined within-person changes pre- and post-diagnosis. Annual differences in sleep-related symptoms between participants with and without incident psychiatric disease were estimated, with positive values indicating worse symptom scores among those who developed psychiatric disease. These models were adjusted for age at year 0, sex, and education attainment. All analyses were performed using SAS software, version 9.4 (SAS Institute Inc, Cary, NC).

## Results

Among 25,038 participants without psychiatric diagnosis at baseline, 716 individuals (2.9%) developed a psychiatric disorder during follow-up (median [IQR] duration, 4 [4–8] years). Compared to those who remained free of psychiatric illness, individuals who later developed a psychiatric condition were younger, more likely to be female, and more often current smokers. They also reported lower levels of physical activity and more frequent use of hypnotic medication. Furthermore, participants with incident psychiatric illness more often had a pre-existing neurological disease. Baseline characteristics for the total population and stratified by incident psychiatric disease are presented in Table 1.

**Table 1:** Characteristics of study population

Characteristics	Total	Incident psychiatry disease		*P* value
		Yes	No	
N	25,038	716	24,322	
Mean age (SD)	51.5 ± 11.5	49.1 ± 11.9	51.6 ± 11.5	<0.0001
Sex				
Female, n (%)	13,750 (54.9)	446 (62.3)	13,304(54.7)	<0.0001
Male, n (%)	11,288 (45.1)	270 (37.7)	11,018(45.3)	
Higher education, n (%)	10,041(40.2)	277 (38.7)	9764 (40.2)	0.4009
Smoking status				
Non-smokers, n (%)	21,830 (88.0)	587 (82.7)	21,243 (88.2)	<0.0001
Current smokers, n (%)	2970 (12.0)	123 (17.3)	2847 (11.8)	
Physical activity				
Low n, (%)	4756 (19.1)	169 (23.7)	4587 (19.0)	0.002
High n, (%)	20,094 (80.9)	543 (76.3)	19,551 (81.0)	
Body mass index				
Underweight, n (%)	221 (0.9)	7 (1.0)	214 (0.9)	0.713
Normal weight, n (%)	11,446 (47.2)	317 (45.5)	11,129 (47.2)	
Overweight, n (%)	9414 (38.8)	272 (39.1)	9142 (38.8)	
Obesity, n (%)	3178 (13.1)	100 (14.4)	3078 (13.1)	
Co-morbidities				
Neurological disease n (%)	2325 (9.3)	91 (12.7)	2234 (9.2)	0.001
Cardiovascular, n (%)	2254 (9.0)	78 (10.9)	2176 (8.9)	0.073
Cancer, n (%)	1521 (6.1)	40 (5.6)	1481 (6.1)	0.579
Hypnotic use				
No, (%)	23,657 (94.5)	619 (86.5)	23,038 (94.7)	<0.0001
Yes, n (%)	1381 (5.5)	97 (13.5)	1284 (5.3)	
Sleep duration				
< 7 h/night, n (%)	4212 (16.8)	128 (17.9)	4084 (16.8)	<0.0001
7–8 h/night, n (%)	10,887 (43.5)	256 (35.8)	10,631 (43.7)	
> 8 h/night, n (%)	9939 (39.7)	332 (46.4)	9607 (39.5)	
Difficulties falling asleep, n (%)	1809 (7.3)	107 (15.1)	1702 (7.1)	<0.0001
Repeated awakenings, n (%)	2472 (10.0)	123 (17.4)	2349 (9.8)	<0.0001
Premature awakening, n (%)	2449 (9.9)	137 (19.4)	2312 (9.6)	<0.0001
Snoring, n (%)	2075 (8.4)	81 (11.4)	1994 (8.3)	0.003
Apnea, n (%)	644 (2.6)	34 (4.9)	610 (2.6)	0.000
Difficulties awakening, n (%)	1442 (5.8)	84 (11.8)	1358 (5.6)	<0.0001
Non-refreshing sleep, n (%)	3881 (15.7)	198 (28.0)	3683 (15.3)	<0.0001
Insufficient sleep, n (%)	2713 (10.9)	137 (19.2)	2576 (10.6)	<0.0001
Persistent fatigue, n (%)	4362 (17.7)	232 (33.0)	4130 (17.2)	<0.0001
Physical exhaustion, n (%)	2549 (10.3)	155 (21.9)	2394 (10.0)	<0.0001
Mental fatigue, n (%)	4161 (16.8)	231 (32.6)	3930 (16.3)	<0.0001

Missing data (n): education (63); smoking status (238); physical activity (188); BMI (779); difficulties falling sleep (199); repeated awakenings (261); premature awakening (362); snoring (399); apnea (580); difficulties awakening (250); non-refreshing sleep (309); insufficient sleep (120); persistent fatigue (327); physical exhaustion (304); mental fatigue (270)

### Associations between individual sleep variables and risk of psychiatric disease

Most baseline sleep characteristics were significantly associated with increased risk of incident psychiatric disease. We observed a U-shaped association between average sleep duration and risk of psychiatric disease (*p* for overall < 0.001, *p* for non-linearity = 0.0007) (eFigure S2). Compared to 7–8 h of sleep per night, both short (< 7 h/night) and long (> 8 h/night) sleep durations were associated with increased risk (HR 1.30, 95% CI 1.04–1.62 and HR 1.40, 95% CI 1.19–1.66, respectively) (Table 2).

**Table 2:** Associations of sleep variables at baseline with incident psychiatry disease

Sleep variable	N	Person-years	Outcome	HR (95% CI)^a^	HR (95% CI)^b^
Sleep duration					
< 7 h	4212	22,661	128	1.37 (1.11–1.70)	1.30 (1.04–1.62)
7–8 h	10,887	61,219	256	1.0 (reference)	1.0 (reference)
> 8 h	9939	55,483	332	1.41 (1.20–1.67)	1.40 (1.19–1.66)
Difficulties falling asleep					
No	23,030	128,303	603	1.0 (reference)	1.0 (reference)
Yes	1809	10,013	107	2.24 (1.82–2.75)	1.63 (1.30–2.04)
Repeated awakenings					
No	22,305	124,236	584	1.0 (reference)	1.0 (reference)
Yes	2472	13,701	123	1.93 (1.58–2.34)	1.55 (1.26–1.91)
Premature awakening					
No	22,227	123,377	570	1.0 (reference)	1.0 (reference)
Yes	2449	14,000	137	2.05 (1.70–2.47)	1.75 (1.44–2.13)
Snoring					
No	22,564	125,506	627	1.0 (reference)	1.0 (reference)
Yes	2075	11,637	81	1.56 (1.23–1.97)	1.27 (0.99–1.63)
Apnea					
No	23,814	132,394	666	1.0 (reference)	1.0 (reference)
Yes	644	3637	34	2.17 (1.53–3.08)	1.69 (1.17–2.45)
Difficulties awakening					
No	23,346	129,848	625	1.0 (reference)	1.0 (reference)
Yes	1442	8179	84	1.90 (1.51–2.40)	1.60 (1.26–2.03)
Non-refreshing sleep					
No	20,848	115,858	510	1.0 (reference)	1.0 (reference)
Yes	3881	21,808	198	1.91 (1.62–2.26)	1.65 (1.39–1.97)
Insufficient sleep					
No	22,205	123,427	577	1.0 (reference)	1.0 (reference)
Yes	2713	15,362	137	1.79 (1.48–2.16)	1.53 (1.26–1.87)
Persistent fatigue					
No	20,349	112,810	472	1.0 (reference)	1.0 (reference)
Yes	4362	24,729	232	2.12 (1.80–2.48)	1.81 (1.53–2.14)
Physical exhaustion					
No	22,185	123,265	554	1.0 (reference)	1.0 (reference)
Yes	2549	14,454	155	2.23 (1.87–2.67)	1.89 (1.56–2.28)
Mental fatigue					
No	20,607	115,961	478	1.0 (reference)	1.0 (reference)
Yes	4161	21,913	231	2.39 (2.03–2.80)	2.09 (1.77–2.48)

^a^Adjusted for age, sex, and education

^b^Adjusted for age, sex, education, smoking status, physical activity, body mass index, hypnotic use, average sleep duration, and comorbidities (cancer, cardiovascular disease, neurological disease); model for sleep duration were not adjusted for average sleep duration

Nighttime insomnia symptoms, including difficulties falling asleep, repeated or premature awakenings, were all associated with elevated risk of incident psychiatric illness, as were non-restorative sleep indicators such as difficulties awakening and non-refreshing sleep. Some of the strongest associations were found for daytime symptoms, including persistent fatigue, physical and mental exhaustion (Table 2). These findings remained largely consistent after excluding the first two years of follow-up (eTable S1) and after stratification by age (eTable S2).

In the basic-adjusted model, we observed a J-shaped association between social jetlag and the risk of incident psychiatric disease, with the lowest risk around 0–1 h of discrepancy between workday and free-day sleep timing. The association remained similar but lost statistical significance after full adjustment for lifestyle and health-related covariates (*p* for overall = 0.149) (eFigure S3).

Mental fatigue emerged as the strongest independent sleep-related predictor of psychiatric disease. In fully adjusted models including all sleep variables, mental fatigue and long sleep duration remained significantly associated with higher risk of incident psychiatric disease (HR 1.66, 95% CI: 1.33–2.09 and HR 1.34, 95% CI 1.14–1.58, respectively) (eTable S3).

### Combined sleep characteristics

Nighttime insomnia at baseline, defined as difficulties falling asleep, repeated awakenings, or premature awakenings occurring at least three times per week, was associated with an increased risk of developing psychiatric disease (HR 1.72, 95% CI 1.45–2.04) (Table 3). Participants reporting either nighttime insomnia or persistent fatigue alone had an elevated risk compared to those without symptoms, while those experiencing both had more than twice the risk of psychiatric disease (HR 2.19, 95% CI 1.77–2.71). A similar pattern was observed when daytime symptoms were defined more broadly as persistent fatigue, physical exhaustion, or mental fatigue.

**Table 3:** Associations between nighttime insomnia, with and without daytime consequences, and incident psychiatry disease

Nighttime insomnia	N		Person-years	Outcome	HR (95% CI)^a^	HR (95% CI)^b^
No	20,409		113,458	487	1.0 (reference)	1.0 (reference)
Yes	4257		23,857	219	**2.09 (1.78–2.46)**	**1.72 (1.45–2.04)**

Nighttime insomnia with or without persistent fatigue						
Nighttime insomnia	Persistent fatigue	N	Person-years	Outcome	HR (95% CI)^a^	HR (95% CI)^b^
No	No	18,121	100,415	382	1.0 (reference)	1.0 (reference)
No	Yes	2160	12,356	100	**1.98 (1.58–2.47)**	**1.77 (1.41–2.22)**
Yes	No	2052	11,460	84	**1.92 (1.51–2.43)**	**1.60 (1.24–2.05)**
Yes	Yes	2156	12,133	132	**2.73 (2.24–3.33)**	**2.19 (1.77–2.71)**

Nighttime insomnia with or without daytime symptoms						
Nighttime insomnia	Daytime symptom	N	Person-years	Outcome	HR (95% CI)^a^	HR (95% CI)^b^
No	No	16,797	93,228	350	1.0 (reference)	1.0 (reference)
No	Yes	3443	19,339	135	**1.72 (1.41–2.10)**	**1.59 (1.29–1.95)**
Yes	No	1587	8911	56	**1.67 (1.26–2.21)**	**1.39 (1.03–1.87)**
Yes	Yes	2621	14,696	160	**2.76 (2.29–3.33)**	**2.24 (1.83–2.74)**

Sleep duration and nighttime insomnia						
Sleep duration	Insomnia	N	Person-years	Outcome	HR (95% CI)^a^	HR (95% CI)^b^
< 7 h	No	3203	17,195	72	1.15 (0.87–1.50)	1.09 (0.83–1.44)
< 7 h	Yes	866	4736	52	**2.93 (2.16–3.99)**	**2.38 (1.72–3.27)**
≥ 7, ≤ 8 h	No	9133	51,176	189	1.0 (reference)	1.0 (reference)
≥ 7, ≤ 8 h	Yes	1652	9454	67	**1.90 (1.43–2.51)**	**1.61 (1.21–2.16)**
> 8 h	No	8073	45,087	226	**1.36 (1.12–1.65)**	**1.38 (1.13–1.68)**
> 8 h	Yes	1739	9667	100	**2.74 (2.14–3.50)**	**2.24 (1.73–2.90)**

Sleep duration and persistent fatigue						
Sleep duration	Persistent fatigue	N	Person-years	Outcome	HR (95% CI)^a^	HR (95% CI)^b^
< 7 h	No	3213	17,121	72	1.18 (0.90–1.55)	1.13 (0.86–1.50)
< 7 h	Yes	915	5050	52	**2.75 (2.02–3.74)**	**2.29 (1.66–3.16)**
≥ 7, ≤ 8 h	No	9037	50,512	181	1.0 (reference)	1.0 (reference)
≥ 7, ≤ 8 h	Yes	1729	10,055	70	**1.83 (1.38–2.41)**	**1.60 (1.20–2.13)**
> 8 h	No	8099	45,177	219	**1.34 (1.10–1.64)**	**1.35 (1.10–1.65)**
> 8 h	Yes	1718	9624	110	**3.00 (2.36–3.81)**	**2.55 (1.99–3.27)**

Sleep duration and daytime symptoms						
Sleep duration	Daytime symptom	N	Person-years	Outcome	HR (95% CI)^a^	HR (95% CI)^b^
< 7 h	No	2886	15,335	63	1.22 (0.91–1.63)	1.16 (0.86–1.57)
< 7 h	Yes	1230	6785	62	**2.57 (1.91–3.45)**	**2.19 (1.62–2.98)**
≥ 7, ≤ 8 h	No	8255	46,220	157	1.0 (reference)	1.0 (reference)
≥ 7, ≤ 8 h	Yes	2490	14,247	95	**1.85 (1.43–2.39)**	**1.67 (1.28–2.17)**
> 8 h	No	7401	41,427	191	**1.35 (1.09–1.67)**	**1.35 (1.09–1.68)**
> 8 h	Yes	2404	13,309	139	**2.86 (2.27–3.61)**	**2.50 (1.97–3.17)**

*Nighttime insomnia* difficulties falling asleep, repeated awakenings and/or premature awakening at least three times per week, *daytime symptoms* persistent fatigue, physical exhaustion, or mental fatigue, *HR* hazard ratio, *CI* confidence interval

^a^Adjusted for age, sex, and education

^b^Adjusted for age, sex, education, smoking status, physical activity, body mass index, hypnotic use, average sleep duration, and comorbidities

Bold values indicate statistical significance

Insomnia and fatigue were consistently associated with elevated risk across all sleep duration groups, with the strongest associations observed in individuals sleeping more than eight hours and reporting either insomnia (HR 2.24, 95% CI 1.73–2.90), persistent fatigue (HR 2.55, 95% CI 1.99–3.27), or daytime symptoms more broadly (HR 2.50, 95% CI 1.97–3.17) (Table 3).

### Longitudinal trajectories of sleep variables before and after incident neurological disease

**Fig. 1:**
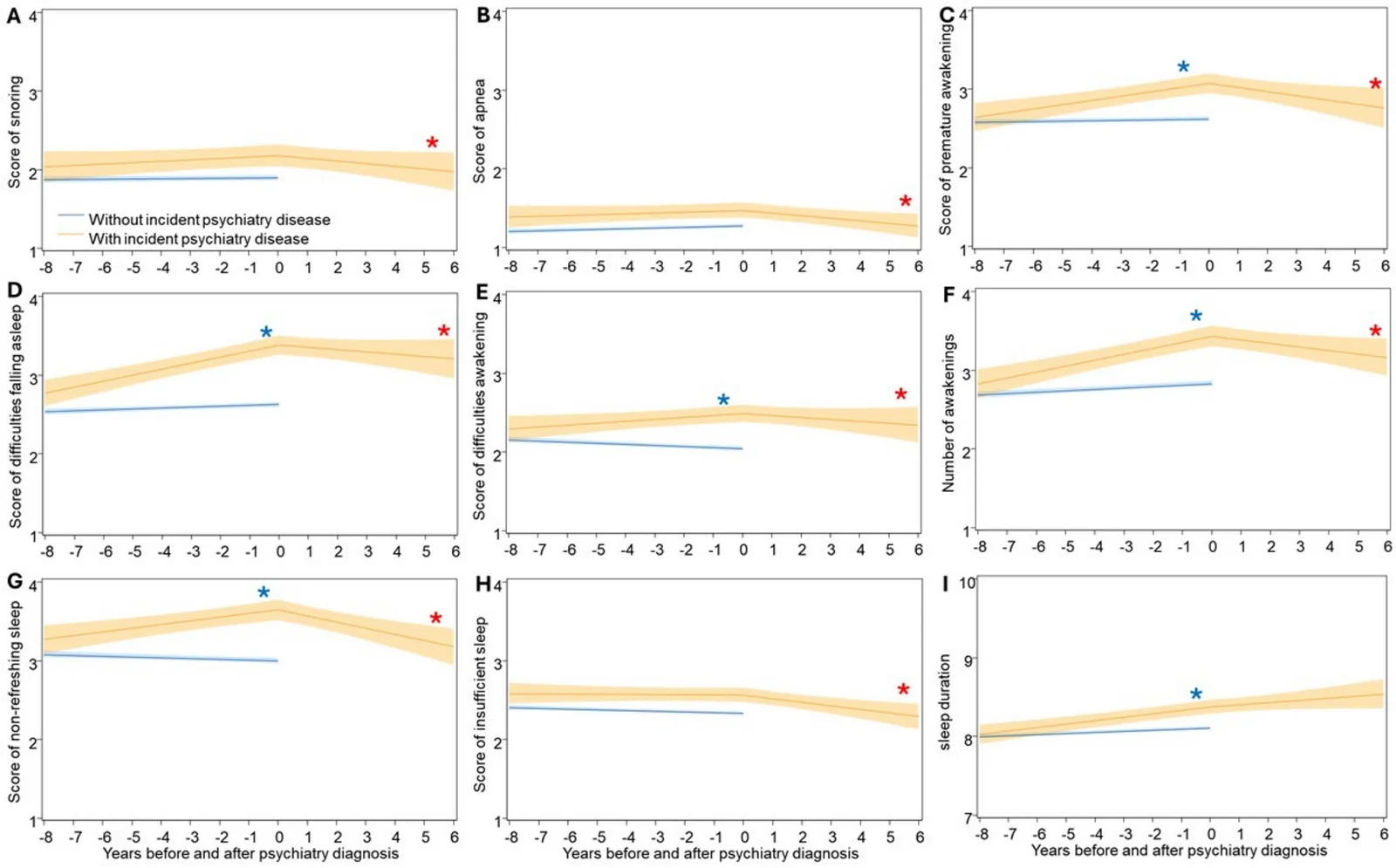
Trajectories of nighttime sleep symptoms before and after psychiatric diagnosis, by disease status. Mean trajectories (solid lines) and 95% confidence intervals (shaded areas) for snoring (**A**), apnea (**B**), premature awakening (**C**), difficulties falling asleep (**D**), difficulties awakening (**E**), number of awakenings (**F**), non-refreshing sleep (**G**), insufficient sleep (**H**), and sleep duration (**I**) were predicted by linear mixed-effect models, adjusted for age at time 0, sex, and education. The retrospective timeline captures the eight years prior to psychiatric diagnosis or end of follow-up. The prospective timeline captures the six years post-diagnosis. The blue star indicates a significant difference in slope before year 0 between those with and without psychiatric disease. The red star indicates a significant difference in slope before and after diagnosis among those with incident psychiatric illness. Details about coefficients are presented in eTable S4

**Fig. 2:**
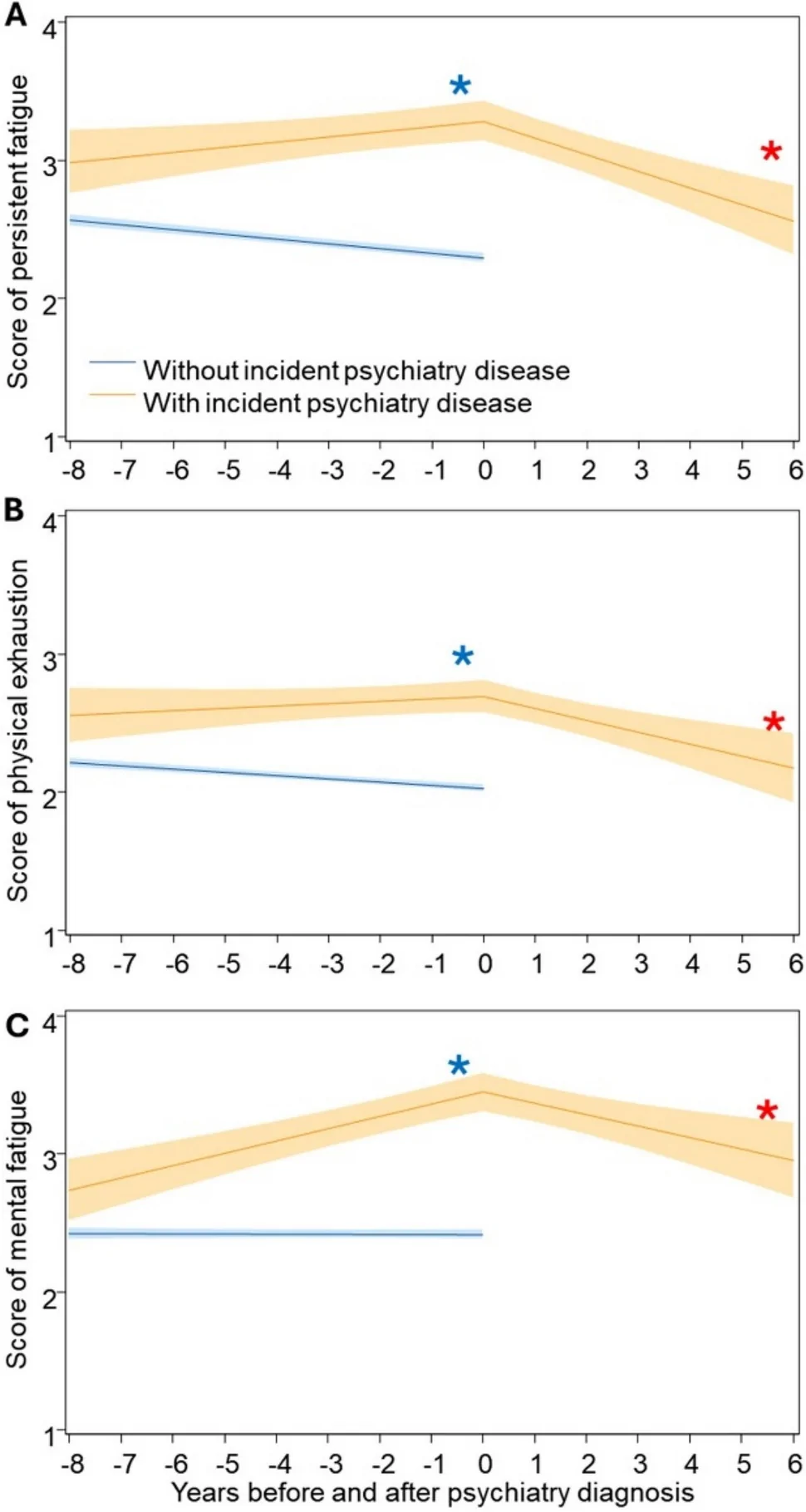
Trajectories of daytime symptoms before and after psychiatric diagnosis, by disease status. Mean trajectories (solid lines) and 95% confidence intervals (shaded areas) for persistent fatigue (**A**), physical exhaustion (**B**), and mental fatigue (**C**) were predicted by linear mixed-effect models, adjusted for age at time 0, sex, and education. The retrospective timeline captures the eight years prior to psychiatric diagnosis or end of follow-up. The prospective timeline captures the six years post-diagnosis. The blue star indicates a significant difference in slope before year 0 between those with and without psychiatric disease. The red star indicates a significant difference in slope before and after diagnosis among those with incident psychiatric illness. Details about coefficients are presented in eTable S4

Pre-diagnostic differences between unaffected individuals and participants who developed psychiatric disease were evident in multiple sleep dimensions (Figs. 1, 2, eTables S4 and S5). Insufficient sleep, snoring, and sleep apnea were more prevalent among those with incident psychiatric disease, with differences remaining relatively stable over time (Fig. 1, eTables S4 and S5). Difficulties falling asleep began to diverge at least eight years prior to diagnosis, while repeated or premature awakenings, difficulties awakening, and non-refreshing sleep began to diverge around 6–7 years prior to diagnosis, with progressively widening differences over time (Fig. 1 and eTable S5). Daytime symptoms, including persistent fatigue, physical exhaustion, and mental fatigue, also differed by psychiatric diseases status from at least eight years prior to diagnosis, with the largest contrasts observed near the time of diagnosis (Fig. 2, eTables S4 and S5).

Most symptom trajectories peaked around the time of diagnosis and showed partial or complete normalization thereafter. For example, the slope of difficulties falling asleep was significantly steeper among participants who developed psychiatric disease compared to unaffected individuals (β = 0.065, 95% CI 0.043–0.086, *p* < 0.0001), followed by a reversal post-diagnosis (Δ slope = −0.105, 95% CI −0.156 to −0.054) (Fig. 1 and eTable S4).

Sleep duration differed modestly between groups, with significant but small increases emerging from year –5 (Fig. 1, eTables S4 and S5) among those who developed psychiatric disease. However, long sleep duration did not show evidence of post-diagnostic improvement (Fig. 1 and eTable S4).

Changes in sleep timing were subtle but detectable in the 5 years leading up to psychiatric diagnosis, with individuals who later developed psychiatric illness gradually waking up later (β difference = 0.036, 95% CI 0.013–0.059, *p* = 0.003) (eTable S5, eFigure S4). We observed no significant differences in social jetlag trajectories between groups (eFigure S5).

## Discussion

In this prospective study, we found that a wide range of sleep-related symptoms, including nighttime insomnia symptoms, sleep-disordered breathing, and daytime dysfunction, were associated with increased risk of subsequent psychiatric disease. These associations were evident several years prior to clinical diagnosis and generally intensified over time. While many symptoms improved after diagnosis, they did not return to the baseline levels observed among unaffected individuals. Long sleep duration and delayed wake-up times continued to worsen post-diagnosis.

These findings indicate that alterations in sleep may not merely reflect secondary effects of emerging psychiatric symptoms but could represent early warning signs of an unfolding disease process. The progressive divergence in sleep-related symptoms between affected and unaffected individuals strengthens the argument for sleep disturbances as part of a prodromal phase, a subtle clinical state that precedes overt psychiatric illness [15–17]. However, the persistent nature of several symptoms following diagnosis complicates a purely prodromal interpretation.

One possible explanation for this pattern is that psychiatric disorders and sleep disturbances share underlying biological mechanisms. Chronic sleep disruption has been associated with increased activity in inflammatory pathways [18], altered hypothalamic–pituitary–adrenal axis function [19], altered monoaminergic signaling [20], and reduced neuroplasticity [21], all of which have also been implicated in the pathophysiology of depression, anxiety, and other psychiatric conditions [22–24]. Shifts in sleep timing, such as progressively later wake-up times, may reflect subtle alterations in circadian regulation. Although we did not observe significant differences in social jetlag, variation in circadian preference and genetic differences in clock genes have been implicated in psychiatric vulnerability [25, 26] and could underlie some of the observed changes.

The strong association between fatigue and subsequent psychiatric illness may also reflect shared biological pathways. Notably, mental fatigue emerged as a stronger predictor of psychiatric disease than several traditional insomnia symptoms. This observation may suggest that impaired daytime functioning reflects emerging psychiatric vulnerability more closely than sleep disturbance itself, although the two are likely closely interconnected. This is supported by longitudinal data and emerging genetic evidence from twin studies suggesting overlapping genetic and environmental factors that predispose individuals to both fatigue and psychiatric disorders [27, 28]. Taken together, the progressive emergence and persistence of symptoms such as fatigue and extended sleep duration in our data may thus reflect neurobiological changes that unfold over time, potentially interacting with psychosocial stressors, genetic predisposition, and emerging psychiatric symptoms in a bidirectional manner.

Not all sleep complaints showed the same temporal patterns. Long sleep duration and mental fatigue were consistently associated with psychiatric onset across subgroups and analytical models and remained elevated post-diagnosis, potentially reflecting enduring neurobiological changes such as persistent inflammation, circadian dysregulation, or medication effects, but could also represent compensatory responses to physiological or emotional strain. In contrast, classical insomnia symptoms were more sensitive to age, follow-up length, and co-occurring symptoms, suggesting heterogeneity in their predictive value. The partial normalization post-diagnosis likely reflects improved symptom management rather than a true resolution of the underlying dysfunction.

From a clinical perspective, the progressive deterioration in sleep before diagnosis could offer opportunities for early detection and intervention. Individuals with cumulative multi-dimensional sleep complaints, particularly when accompanied by fatigue or sleep timing shifts, could be at elevated risk of psychiatric illness. Monitoring such patterns over time might help identify individuals in the early stages of disease development, potentially enabling targeted support before the onset of full clinical symptoms.

Strengths of this study include its large sample, repeated assessments over a long follow-up period, and the wide range of sleep variables examined. The use of a backward timescale allowed us to examine changes in sleep relative to the timing of diagnosis, improving temporal resolution. However, several limitations should be considered. Respondents were more often women, older, born in Sweden, and more likely to hold a university degree, limiting generalizability beyond the Swedish working-age population. Trajectory analyses required participation in three or more waves, which further selects for more stably engaged participants. However, the baseline prevalence of sleep-related complaints was similar in the total eligible sample and in the subset contributing three or more waves, which mitigates the risk of selection bias. All sleep variables were self-reported, and although this enhances ecological validity, it may introduce differential misclassification that would dilute associations. Information regarding objective sleep apnea diagnosis, severity, and treatment was not available. Future studies incorporating clinical sleep assessments and treatment data are needed to determine whether effective treatment of sleep-disordered breathing influences the subsequent risk of psychiatric illness. Diagnostic classification relied on national registers covering inpatient and specialized and outpatient care and the Cause of Death Register and may not capture subthreshold or undiagnosed psychiatric conditions. Although we captured diagnoses from 1997 onward and excluded individuals with a recorded psychiatric diagnosis at baseline, some participants may have had diagnoses prior to 1997, with subsequent remission, leading to misclassification of prior cases as incident. Information on specific psychiatric diagnoses was not available in the linked dataset. Consequently, we were unable to investigate whether the observed associations differed across psychiatric diagnostic categories. Finally, despite adjustment for a broad set of covariates, residual and time-varying confounding remain possible.

In conclusion, changes in sleep patterns and daytime functioning appear to emerge several years prior to psychiatric diagnosis, with many symptoms stabilizing or partially improving thereafter. While a causal role for sleep disturbances cannot be excluded, the overall temporal pattern aligns more closely with a prodromal phase of psychiatric disease. In settings where repeated assessment is feasible, sleep-related symptoms may serve as valuable early warning signs.

## Supplementary Information

Below is the link to the electronic supplementary material.

Supplementary file1 (DOCX 749 KB)

## Data Availability

The SLOSH data generated and analyzed during the current study is not publicly available due to ethical restrictions and considering that sensitive personal data are involved. Access to the data may be provided to other researchers in line with Swedish law and after consultation with the Stockholm University legal department. Requests for SLOSH data, stored at the Department of Psychology, Stockholm University should be sent to [data@slosh.se](mailto:data@slosh.se).
