# Supplementary material for: Sleep disturbances as early indicators of incident psychiatric disease
Source: Eur Arch Psychiatry Clin Neurosci. 2026 Aug 11;276(7):3715–24. doi: 10.1007/s00406-026-02332-9 (PMC13562121; doi:10.1007/s00406-026-02332-9)
Supplement: Supplementary file 1 [file 406_2026_2332_MOESM1_ESM.docx]

**eTable 1. Associations of sleep variables with incident psychiatric disease after excluding the first two years follow-up.**

| Sleep variable | | HR (95% CI) |
| --- | --- | --- |
| Sleep duration | <7 h | 1.41 (1.07-1.84) |
|  | ≥7, ≤8 h | 1.0 (reference) |
|  | >8 h | 1.39 (1.12-1.71) |
| Difficulties falling asleep | No | 1.0 (reference) |
|  | Yes | 1.77 (1.34-2.35) |
| Repeated awakenings | No | 1.0 (reference) |
|  | Yes | 1.43 (1.09-1.87) |
| Premature awakening | No | 1.0 (reference) |
|  | Yes | 1.46 (1.13-1.89) |
| Snoring | No | 1.0 (reference) |
|  | Yes | 1.08 (0.77-1.50) |
| Apnea | No | 1.0 (reference) |
|  | Yes | 1.42 (0.86-2.33) |
| Difficulties awakening | No | 1.0 (reference) |
|  | Yes | 1.57 (1.16-2.12) |
| Non-refreshing sleep | No | 1.0 (reference) |
|  | Yes | 1.47 (1.18-1.84) |
| Insufficient sleep | No | 1.0 (reference) |
|  | Yes | 1.35 (1.04-1.75) |
| Persistent fatigue | No | 1.0 (reference) |
|  | Yes | 1.72 (1.39-2.13) |
| Physical exhaustion | No | 1.0 (reference) |
|  | Yes | 1.78 (1.40-2.26) |
| Mental fatigue | No | 1.0 (reference) |
|  | Yes | 1.90 (1.53-2.36) |

Adjusted for age, sex, education, smoking status, physical activity, body mass index, hypnotic use, average sleep duration, and comorbidities (cancer, psychiatric illness, cardiovascular disease); model for sleep duration was not adjusted for average sleep duration.

**eTable 2. Associations of sleep variables with incident psychiatric disease in age-specific groups.**

|  |  | HR (95% CI) |  |  |
| --- | --- | --- | --- | --- |
| Sleep variable | | <40 years | 40-60 years | ≥60 years |
| Sleep duration | <7 h | 1.34 (0.84-2.10) | 1.26 (0.94-1.69) | 1.35 (0.82-2.18) |
|  | 7-8 h | 1.0 (reference) | 1.0 (reference) | 1.0 (reference) |
|  | >8 h | 1.38 (0.98-1.97) | 1.38 (1.09-1.74) | 1.47 (1.04-2.11) |
| Difficulties falling asleep | No | 1.0 (reference) | 1.0 (reference) | 1.0 (reference) |
|  | Yes | 2.20 (1.39-3.37) | 1.36 (0.97-1.87) | 1.82 (1.12-2.85) |
| Repeated awakenings | No | 1.0 (reference) | 1.0 (reference) | 1.0 (reference) |
|  | Yes | 1.38 (0.81-2.22) | 1.61 (1.21-2.11) | 1.55 (0.99-2.33) |
| Premature awakening | No | 1.0 (reference) | 1.0 (reference) | 1.0 (reference) |
|  | Yes | 1.27 (0.81-1.93) | 2.02 (1.56-2.59) | 1.65 (1.02-2.55) |
| Snoring | No | 1.0 (reference) | 1.0 (reference) | 1.0 (reference) |
|  | Yes | 1.20 (0.58-2.23) | 1.32 (0.94-1.83) | 1.24 (0.77-1.92) |
| Apnea | No | 1.0 (reference) | 1.0 (reference) | 1.0 (reference) |
|  | Yes | 2.98 (1.23-6.13) | 1.32 (0.71-2.24) | 1.74 (0.90-3.08) |
| Difficulties awakening | No | 1.0 (reference) | 1.0 (reference) | 1.0 (reference) |
|  | Yes | 2.08 (1.43-2.98) | 1.38 (0.96-1.92) | 0.81 (0.25-1.93) |
| Non-refreshing sleep | No | 1.0 (reference) | 1.0 (reference) | 1.0 (reference) |
|  | Yes | 1.54 (1.10-2.13) | 1.89 (1.49-2.38) | 1.15 (0.70-1.80) |
| Insufficient sleep | No | 1.0 (reference) | 1.0 (reference) | 1.0 (reference) |
|  | Yes | 1.49 (1.01-2.16) | 1.57 (1.20-2.04) | 1.50 (0.87-2.44) |
| Persistent fatigue | No | 1.0 (reference) | 1.0 (reference) | 1.0 (reference) |
|  | Yes | 1.71 (1.22-2.38) | 1.97 (1.57-2.46) | 1.52 (1.00-2.24) |
| Physical exhaustion | No | 1.0 (reference) | 1.0 (reference) | 1.0 (reference) |
|  | Yes | 1.72 (1.18-2.46) | 2.04 (1.58-2.62) | 1.69 (1.04-2.63) |
| Mental fatigue | No | 1.0 (reference) | 1.0 (reference) | 1.0 (reference) |
|  | Yes | 1.80 (1.28-2.50) | 2.27 (1.82-2.84) | 1.97 (1.27-2.95) |

Adjusted for age, sex, education, smoking status, physical activity, body mass index, hypnotic use, average sleep duration, and comorbidities (cancer, psychiatric illness, cardiovascular disease); model for sleep duration was not adjusted for average sleep duration.

**eTable 3. Associations of sleep variables with incident psychiatric disease: multi-adjusted for sleep variables.**

| Sleep variable | | HR (95% CI) |
| --- | --- | --- |
| Sleep duration | <7 h | 1.10 (0.80-1.53) |
|  | 7-8 h | 1.0 (reference) |
|  | >8 h | **1.34 (1.14-1.58)** |
| Difficulties falling asleep | No | 1.0 (reference) |
|  | Yes | 1.08 (0.82-1.43) |
| Repeated awakenings | No | 1.0 (reference) |
|  | Yes | 0.96 (0.73-1.27) |
| Premature awakening | No | 1.0 (reference) |
|  | Yes | 1.25 (0.96-1.62) |
| Snoring | No | 1.0 (reference) |
|  | Yes | 1.00 (0.74-1.35) |
| Apnea | No | 1.0 (reference) |
|  | Yes | 1.42 (0.93-2.19) |
| Difficulties awakening | No | 1.0 (reference) |
|  | Yes | 1.18 (0.90-1.55) |
| Non-refreshing sleep | No | 1.0 (reference) |
|  | Yes | 1.00 (0.77-1.28) |
| Insufficient sleep | No | 1.0 (reference) |
|  | Yes | 1.07 (0.83-1.36) |
| Persistent fatigue | No | 1.0 (reference) |
|  | Yes | 1.13 (0.88-1.44) |
| Physical exhaustion | No | 1.0 (reference) |
|  | Yes | 1.20 (0.95-1.52) |
| Mental fatigue | No | 1.0 (reference) |
|  | Yes | **1.66 (1.33-2.09)** |

Adjusted for age, sex, education, smoking status, physical activity, body mass index, hypnotic use, average sleep duration, comorbidities (cancer, neurology disease, cardiovascular disease), and all sleep variables in the table.

**eTable 4. β coefficient and 95% confidence interval (CI) of sleep-related symptom trajectories before and after psychiatric diagnosis.**

| Sleep-related symptom | β coefficient (95% CI) | *P-value* |
| --- | --- | --- |
| **Sleep duration** |  |  |
| Slope before year 0 for psychiatry-free subjects | -0.086 (-0.097, -0.075) | <.0001 |
| Slope before year 0 for incident psychiatry subjects | -0.056 (-0.074, -0.038) | <.0001 |
| Difference in slope before year 0 | **0.030 (0.015, 0.045)** | <.0001 |
| Slope after year 0 for incident psychiatry subjects | -0.073 (-0.104, -0.041) | <.0001 |
| Difference in slope before and after year 0 | -0.017 (-0.053, 0.020) | 0.366 |
| **Difficulties falling asleep** |  |  |
| Slope before year 0 for psychiatry-free subjects | 0.024 (0.008, 0.040) | 0.003 |
| Slope before year 0 for incident psychiatry subjects | 0.088 (0.063, 0.114) | <.0001 |
| Difference in slope before year 0 | **0.065 (0.043, 0.086)** | <.0001 |
| Slope after year 0 for incident psychiatry subjects | -0.017 (-0.060, 0.027) | 0.458 |
| Difference in slope before and after year 0 | **-0.105 (-0.156, -0.054)** | <.0001 |
| **Repeated awakenings** |  |  |
| Slope before year 0 for psychiatry-free subjects | 0.072 (0.055, 0.089) | <.0001 |
| Slope before year 0 for incident psychiatry subjects | 0.130 (0.102, 0.157) | <.0001 |
| Difference in slope before year 0 | **0.058 (0.034, 0.081)** | <.0001 |
| Slope after year 0 for incident psychiatry subjects | 0.009 (-0.032, 0.051) | 0.658 |
| Difference in slope before and after year 0 | **-0.120 (-0.171, -0.069)** | <.0001 |
| **Premature awakening** |  |  |
| Slope before year 0 for psychiatry-free subjects | 0.072 (0.054, 0.090) | <.0001 |
| Slope before year 0 for incident psychiatry subjects | 0.121 (0.092, 0.151) | <.0001 |
| Difference in slope before year 0 | **0.049 (0.025, 0.074)** | <.0001 |
| Slope after year 0 for incident psychiatry subjects | 0.014 (-0.032, 0.060) | 0.545 |
| Difference in slope before and after year 0 | **-0.107 (-0.162, -0.052)** | 0.000 |
| **Snoring** |  |  |
| Slope before year 0 for psychiatry-free subjects | 0.077 (0.059, 0.094) | <.0001 |
| Slope before year 0 for incident psychiatry subjects | 0.092 (0.063, 0.121) | <.0001 |
| Difference in slope before year 0 | 0.016 (-0.009, 0.040) | 0.217 |
| Slope after year 0 for incident psychiatry subjects | 0.040 (-0.003, 0.082) | 0.067 |
| Difference in slope before and after year 0 | **-0.052 (-0.104, -0.001)** | 0.047 |
| **Apnea** |  |  |
| Slope before year 0 for psychiatry-free subjects | 0.031 (0.019, 0.044) | <.0001 |
| Slope before year 0 for incident psychiatry subjects | 0.033 (0.013, 0.054) | 0.002 |
| Difference in slope before year 0 | 0.002 (-0.016, 0.019) | 0.839 |
| Slope after year 0 for incident psychiatry subjects | -0.011 (-0.037, 0.016) | 0.427 |
| Difference in slope before and after year 0 | **-0.044 (-0.077, -0.011)** | 0.010 |
| **Difficulties awakening** |  |  |
| Slope before year 0 for psychiatry-free subjects | 0.002 (-0.013, 0.018) | 0.765 |
| Slope before year 0 for incident psychiatry subjects | 0.040 (0.016, 0.065) | 0.001 |
| Difference in slope before year 0 | **0.038 (0.017, 0.059)** | 0.000 |
| Slope after year 0 for incident psychiatry subjects | -0.008 (-0.048, 0.032) | 0.706 |
| Difference in slope before and after year 0 | **-0.048 (-0.095, -0.001)** | 0.046 |
| **Non-refreshing sleep** |  |  |
| Slope before year 0 for psychiatry-free subjects | 0.025 (0.008, 0.043) | 0.005 |
| Slope before year 0 for incident psychiatry subjects | 0.082 (0.053, 0.111) | <.0001 |
| Difference in slope before year 0 | **0.057 (0.033, 0.081)** | <.0001 |
| Slope after year 0 for incident psychiatry subjects | -0.043 (-0.085, -0.002) | 0.041 |
| Difference in slope before and after year 0 | **-0.125 (-0.176, -0.075)** | <.0001 |
| **Insufficient sleep** |  |  |
| Slope before year 0 for psychiatry-free subjects | 0.023 (0.011, 0.036) | 0.000 |
| Slope before year 0 for incident psychiatry subjects | 0.030 (0.010, 0.051) | 0.003 |
| Difference in slope before year 0 | 0.007 (-0.010, 0.024) | 0.418 |
| Slope after year 0 for incident psychiatry subjects | -0.013 (-0.041, 0.016) | 0.375 |
| Difference in slope before and after year 0 | **-0.043 (-0.078, -0.009)** | 0.015 |
| **Persistent fatigue** |  |  |
| Slope before year 0 for psychiatry-free subjects | -0.023 (-0.040, -0.006) | 0.009 |
| Slope before year 0 for incident psychiatry subjects | 0.048 (0.014, 0.082) | 0.006 |
| Difference in slope before year 0 | **0.071 (0.041, 0.101)** | <.0001 |
| Slope after year 0 for incident psychiatry subjects | -0.110 (-0.155, -0.065) | <.0001 |
| Difference in slope before and after year 0 | **-0.157 (-0.215, -0.099)** | <.0001 |
| **Physical exhaustion** |  |  |
| Slope before year 0 for psychiatry-free subjects | -0.049 (-0.065, -0.034) | <.0001 |
| Slope before year 0 for incident psychiatry subjects | -0.009 (-0.039, 0.022) | 0.566 |
| Difference in slope before year 0 | **0.041 (0.014, 0.068)** | 0.003 |
| Slope after year 0 for incident psychiatry subjects | -0.112 (-0.158, -0.067) | <.0001 |
| Difference in slope before and after year 0 | **-0.104 (-0.160, -0.047)** | 0.000 |
| **Mental fatigue** |  |  |
| Slope before year 0 for psychiatry-free subjects | 0.042 (0.025, 0.060) | <.0001 |
| Slope before year 0 for incident psychiatry subjects | 0.132 (0.098, 0.165) | <.0001 |
| Difference in slope before year 0 | **0.089 (0.059, 0.119)** | <.0001 |
| Slope after year 0 for incident psychiatry subjects | -0.040 (-0.089, 0.010) | 0.115 |
| Difference in slope before and after year 0 | **-0.171 (-0.233, -0.109)** | <.0001 |

Year 0 was defined as the year of diagnosis for cases or the end of follow-up for those without psychiatry disease. Models were adjusted for age at time 0, sex, and education.

**eTable 5. Differences in sleep variables with 95% confidence intervals (CI) between individuals who developed psychiatric disease and those who remained unaffected during the eight years preceding diagnosis.**

|  | Difficulties falling asleep | | | Repeated awakenings | | | | Premature awakening | | | | Snoring | |  | | |
| --- | --- | --- | --- | --- | --- | --- | --- | --- | --- | --- | --- | --- | --- | --- | --- | --- |
| Year | Mean difference (95% CI) | P-value | Mean difference (95% CI) | | P-value | Mean difference (95% CI) | | | P-value | Mean difference (95% CI) | | | P-value | |  |  |
| -8 | **0.24 (0.04, 0.43)** | 0.005 | 0.14 (-0.07, 0.35) | | 0.122 | 0.06 (-0.15, 0.27) | | | 0.509 | 0.17 (-0.06, 0.39) | | | 0.095 | |  |  |
| -7 | **0.3 (0.13, 0.48)** | <.0001 | **0.20 (0.01, 0.39)** | | 0.018 | 0.11 (-0.08, 0.30) | | | 0.195 | 0.18 (-0.03, 0.39) | | | 0.049 | |  |  |
| -6 | **0.37 (0.21, 0.53)** | <.0001 | **0.25 (0.08, 0.43)** | | 0.001 | 0.16 (-0.01, 0.33) | | | 0.038 | **0.20 (0.01, 0.38)** | | | 0.020 | |  |  |
| -5 | **0.43 (0.28, 0.58)** | <.0001 | **0.31 (0.16, 0.47)** | | <.0001 | **0.21 (0.05, 0.36)** | | | 0.002 | **0.21 (0.04, 0.38)** | | | 0.007 | |  |  |
| -4 | **0.50 (0.36, 0.63)** | <.0001 | **0.37 (0.22, 0.52)** | | <.0001 | **0.26 (0.12, 0.40)** | | | <.0001 | **0.23 (0.07, 0.39)** | | | 0.002 | |  |  |
| -3 | **0.56 (0.43, 0.69)** | <.0001 | **0.43 (0.29, 0.57)** | | <.0001 | **0.31 (0.17, 0.44)** | | | <.0001 | **0.24 (0.09, 0.40)** | | | 0.001 | |  |  |
| -2 | **0.63 (0.5, 0.76)** | <.0001 | **0.48 (0.34, 0.62)** | | <.0001 | **0.36 (0.22, 0.49)** | | | <.0001 | **0.26 (0.11, 0.41)** | | | 0.000 | |  |  |
| -1 | **0.69 (0.56, 0.82)** | <.0001 | **0.54 (0.40, 0.69)** | | <.0001 | **0.41 (0.27, 0.54)** | | | <.0001 | **0.27 (0.12, 0.43)** | | | 0.000 | |  |  |
| 0 | **0.75 (0.61, 0.9)** | <.0001 | **0.60 (0.45, 0.75)** | | <.0001 | **0.45 (0.31, 0.60)** | | | <.0001 | **0.29 (0.13, 0.45)** | | | 0.000 | |  |  |
|  | Apnoea |  | Difficulties awakening | | | | Non-refreshing sleep | | | | Insufficient sleep | | | | |  |
| Year | Mean difference (95% CI) | P-value | Mean difference (95% CI) | | P-value | Mean difference (95% CI) | | | P-value | Mean difference (95% CI) | | | P-value | |  |  |
| -8 | **0.18 (0.02, 0.35)** | 0.010 | 0.14 (-0.04, 0.33) | | 0.074 | 0.19 (-0.02, 0.4) | | | 0.037 | **0.18 (0.03, 0.33)** | | | 0.006 | |  |  |
| -7 | **0.19 (0.04, 0.33)** | 0.005 | **0.18 (0.01, 0.35)** | | 0.014 | **0.25 (0.06, 0.44)** | | | 0.003 | **0.19 (0.05, 0.32)** | | | 0.002 | |  |  |
| -6 | **0.19 (0.05, 0.32)** | 0.002 | **0.22 (0.07, 0.37)** | | 0.001 | **0.30 (0.13, 0.48)** | | | <.0001 | **0.19 (0.07, 0.32)** | | | 0.000 | |  |  |
| -5 | **0.19 (0.07, 0.31)** | 0.001 | **0.26 (0.12, 0.40)** | | <.0001 | **0.36 (0.20, 0.52)** | | | <.0001 | **0.20 (0.09, 0.31)** | | | <.0001 | |  |  |
| -4 | **0.19 (0.08, 0.31)** | 0.001 | **0.30 (0.17, 0.42)** | | <.0001 | **0.42 (0.27, 0.56)** | | | <.0001 | **0.21 (0.10, 0.31)** | | | <.0001 | |  |  |
| -3 | **0.19 (0.08, 0.30)** | 0.000 | **0.33 (0.21, 0.46)** | | <.0001 | **0.47 (0.34, 0.61)** | | | <.0001 | **0.22 (0.12, 0.31)** | | | <.0001 | |  |  |
| -2 | **0.19 (0.09, 0.30)** | 0.000 | **0.37 (0.25, 0.49)** | | <.0001 | **0.53 (0.39, 0.67)** | | | <.0001 | **0.22 (0.13, 0.32)** | | | <.0001 | |  |  |
| -1 | **0.20 (0.09, 0.30)** | 0.000 | **0.41 (0.29, 0.53)** | | <.0001 | **0.59 (0.45, 0.73)** | | | <.0001 | **0.23 (0.13, 0.33)** | | | <.0001 | |  |  |
| 0 | **0.20 (0.08, 0.31)** | 0.000 | **0.45 (0.32, 0.58)** | | <.0001 | **0.64 (0.49, 0.8)** | | | <.0001 | **0.24 (0.13, 0.34)** | | | <.0001 | |  |  |
|  | Persistent fatigue |  | Physical exhaustion | |  | Mental fatigue | | |  | Sleep duration | | |  | |  |  |
| Year | Mean difference (95% CI) | P-value | Mean difference (95% CI) | | P-value | Mean difference (95% CI) | | | P-value | Mean difference (95% CI) | | | P-value | |  |  |
| -8 | **0.42 (0.15, 0.69)** | 0.000 | **0.34 (0.11, 0.57)** | | 0.001 | **0.32 (0.05, 0.58)** | | | 0.005 | 0.03 (-0.11, 0.17) | | | 0.656 | |  |  |
| -7 | **0.49 (0.25, 0.73)** | <.0001 | **0.38 (0.17, 0.59)** | | <.0001 | **0.41 (0.17, 0.64)** | | | <.0001 | 0.06 (-0.07, 0.19) | | | 0.322 | |  |  |
| -6 | **0.56 (0.35, 0.78)** | <.0001 | **0.42 (0.24, 0.60)** | | <.0001 | **0.50 (0.29, 0.70)** | | | <.0001 | 0.09 (-0.03, 0.21) | | | 0.101 | |  |  |
| -5 | **0.63 (0.44, 0.83)** | <.0001 | **0.46 (0.30, 0.62)** | | <.0001 | **0.58 (0.4, 0.77)** | | | <.0001 | **0.12 (0.01, 0.23)** | | | 0.017 | |  |  |
| -4 | **0.70 (0.53, 0.88)** | <.0001 | **0.50 (0.36, 0.64)** | | <.0001 | **0.67 (0.51, 0.84)** | | | <.0001 | **0.15 (0.05, 0.25)** | | | 0.001 | |  |  |
| -3 | **0.78 (0.61, 0.94)** | <.0001 | **0.54 (0.41, 0.67)** | | <.0001 | **0.76 (0.61, 0.92)** | | | <.0001 | **0.18 (0.08, 0.28)** | | | <.0001 | |  |  |
| -2 | **0.85 (0.69, 1.00)** | <.0001 | **0.58 (0.46, 0.71)** | | <.0001 | **0.85 (0.7, 1.00)** | | | <.0001 | **0.21 (0.11, 0.30)** | | | <.0001 | |  |  |
| -1 | **0.92 (0.76, 1.08)** | <.0001 | **0.62 (0.50, 0.75)** | | <.0001 | **0.94 (0.79, 1.09)** | | | <.0001 | **0.24 (0.14, 0.34)** | | | <.0001 | |  |  |
| 0 | **0.99 (0.82, 1.16)** | <.0001 | **0.66 (0.53, 0.80)** | | <.0001 | **1.03 (0.87, 1.2)** | | | <.0001 | **0.27 (0.17, 0.37)** | | | <.0001 | |  |  |

Differences in sleep variables were calculated as the mean value in participants with incident psychiatry disease minus that in those who remained free of psychiatry disease. Positive values indicate longer sleep duration or more severe nighttime or daytime symptoms in participants who later developed psychiatry diseases. Estimates were adjusted for age at time 0, sex, and education.

**eTable 6. β coefficient and 95% confidence interval (CI) of sleep timing trajectories before and after diagnosis of psychiatry disease.**

| **Sleep timing** | **β coefficient (95% CI)** | ***P-value*** |
| --- | --- | --- |
| **Workday bedtime** |  |  |
| Slope before year 0 for neurology-free subjects | -0.096 (-0.106, -0.086) | <.0001 |
| Slope before year 0 for incident neurology subjects | -0.087 (-0.103, -0.071) | <.0001 |
| Difference in slope before year 0 | 0.009 (-0.004, 0.023) | 0.187 |
| Slope after year 0 for incident neurology subjects | -0.074 (-0.104, -0.044) | <.0001 |
| Difference in slope before and after year 0 | 0.013 (-0.021, 0.047) | 0.460 |
| **Workday wake-up time** |  |  |
| Slope before year 0 for neurology-free subjects | -0.195 (-0.214, -0.176) | <.0001 |
| Slope before year 0 for incident neurology subjects | -0.165 (-0.196, -0.135) | <.0001 |
| Difference in slope before year 0 | **0.029 (0.004, 0.055)** | **0.023** |
| Slope after year 0 for incident neurology subjects | -0.146 (-0.194, -0.098) | <.0001 |
| Difference in slope before and after year 0 | 0.019 (-0.039, 0.077) | 0.516 |
| **Free day bedtime** |  |  |
| Slope before year 0 for neurology-free subjects | -0.092 (-0.103, -0.082) | <.0001 |
| Slope before year 0 for incident neurology subjects | -0.079 (-0.097, -0.062) | <.0001 |
| Difference in slope before year 0 | 0.013 (-0.001, 0.028) | 0.075 |
| Slope after year 0 for incident neurology subjects | -0.087 (-0.118, -0.057) | <.0001 |
| Difference in slope before and after year 0 | -0.008 (-0.043, 0.027) | 0.641 |
| **Free day wake-up time** |  |  |
| Slope before year 0 for neurology-free subjects | -0.070 (-0.087, -0.053) | <.0001 |
| Slope before year 0 for incident neurology subjects | -0.034 (-0.062, -0.007) | 0.015 |
| Difference in slope before year 0 | **0.036 (0.013, 0.059)** | **0.003** |
| Slope after year 0 for incident neurology subjects | -0.044 (-0.087, -0.001) | 0.047 |
| Difference in slope before and after year 0 | -0.009 (-0.062, 0.043) | 0.727 |
| **Social jetlag** |  |  |
| Slope before year 0 for neurology-free subjects | 0.080 (0.069, 0.091) | <.0001 |
| Slope before year 0 for incident neurology subjects | 0.073 (0.056, 0.091) | <.0001 |
| Difference in slope before year 0 | -0.007 (-0.021, 0.008) | 0.382 |
| Slope after year 0 for incident neurology subjects | 0.063 (0.028, 0.097) | 0.000 |
| Difference in slope before and after year 0 | -0.011 (-0.049, 0.028) | 0.590 |

Year 0 was defined as the year of diagnosis for cases or the end of follow-up for those without psychiatry disease. Models were adjusted for age at time 0, sex, and education.

**eFigure 1. Flow chart of study population**

29,651 participants included in the SLOSH since 2010

25,038 participants with sleep data and free of psychiatric disease

Excluding 2781 participants with prevalent psychiatric disease

Excluding 1832 participants without any sleep data

**eFigure 2. Associations between average sleep duration and risk of psychiatric disease, multi-adjusted model**

Model adjusted for age, sex, education, smoking status, physical activity, body mass index, hypnotic use, average sleep duration, and comorbidities, p for overall <0.001, p for non-linear=0.0007.

**eFigure 3. Associations between social jetlag and risk of psychiatric disease, basic- and multi-adjusted model.**

Social jetlag was defined as the difference between the midpoint of sleep on free days and workdays. Hazards ratios with 95% confidence intervals were calculated using Cox regression models with restricted cubic splines. Basic-adjusted model (A) included age, sex, and education. Multi-adjusted model (B) included age, sex, education, smoking status, physical activity, body mass index, hypnotic use, and comorbidities.

**eFigure 4. Trajectories of sleep timing on workdays and free days before and after psychiatric diagnosis, by psychiatry disease status.**

Mean trajectories (solid lines) and 95% confidence intervals (shaded areas) for bed timing and wake-up timing on workdays were predicted by linear mixed-effect models, adjusted for age at time 0, sex, and education. The retrospective timeline captures the eight years prior to psychiatry disease diagnosis or end of follow-up. The prospective timeline captures the six years after the diagnosis. Details about coefficients are presented in **eTable 5**.

**eFigure 5. Trajectories of social jet lag before and after psychiatric diagnosis, by psychiatry disease status.**

Mean trajectories (solid lines) and 95% confidence intervals (shaded areas) for bed timing and wake-up timing on workdays were predicted by linear mixed-effect models, adjusted for age at time 0, sex, and education. The retrospective timeline captures the eight years prior to psychiatry disease diagnosis or end of follow-up. The prospective timeline captures the six years after the diagnosis. Details about coefficients are presented in **eTable 5**.
